# Ag/Cr-TiO_2_ and Pd/Cr-TiO_2_ for Organic Dyes Elimination and Treatment of Polluted River Water in Presence of Visible Light

**DOI:** 10.3390/nano13162341

**Published:** 2023-08-15

**Authors:** Mariana Alejandra Gil, Julie J. Murcia, Mónica Hernández-Laverde, Nicola Morante, Diana Sannino, Vincenzo Vaiano

**Affiliations:** 1 Grupo de Catálisis, Universidad Pedagógica y Tecnológica de Colombia UPTC, Avenida Central del Norte, Tunja 150002, Boyacá, Colombia; mariana.gil@uptc.edu.co (M.A.G.); julie.murcia@uptc.edu.co (J.J.M.); monica.hernandez06@uptc.edu.co (M.H.-L.); 2 Grupo GIA UNAD, Escuela de Ciencias Básicas Tecnología e Ingeniería, Universidad Nacional Abierta y a Distancia UNAD, Sogamoso 152217, Boyacá, Colombia; 3 Department of Industrial Engineering, University of Salerno, Via Giovanni Paolo II, 132, 84084 Fisciano, Italy; nmorante@unisa.it (N.M.); vvaiano@unisa.it (V.V.)

**Keywords:** visible light photocatalysts, noble metals decorated TiCrOx photocatalysts, dye, bacteria elimination

## Abstract

In this work, photocatalytic materials constituted by Cr-doped TiO_2_ (Cr-TiO_2_) decorated with noble metals show high effectiveness in the mineralization of Acid Orange 7 (AO7) and in the disinfection of real river water. The materials were firstly obtained by sol-gel method to get Cr-TiO_2_ that was subsequently modified by photochemical deposition of Ag or Pd nanoparticles (Ag/Cr-TiO_2_, Pd/Cr-TiO_2_). Chemical-physical characterization results evidenced that the noble metals were homogeneously distributed on the Cr-TiO_2_ surface. By using Pd(0.25%)/Cr-TiO_2_, the AO7 discoloration efficiency was about 91.4% after only 60 min of visible irradiation, which can be due to the lowest band gap of this material. Moreover, nitrates, chlorides, total hardness, and coliform bacteria content significantly decreased after the treatment of real river water samples (that is contaminated by industrial and domestic effluents) under UV and visible light irradiation in the presence of TiCrOx decorated with noble metals. One hundred percent of elimination rate for *E. coli*, total coliforms, and other enterobacteriaceae (without regrowth) was achieved by using Ag/Cr-TiO_2_ as photocatalyst.

## 1. Introduction

Nanomaterials based on TiO_2_ have been extensively evaluated in the removal of metals, in the elimination of microorganisms and in the photodegradation of complex pollutants in liquid and gas phases. By heterogeneous photocatalysis processes, the treatment of wastewater and natural water sources is achieved. Despite the advantages of TiO_2_ as photocatalyst, the intrinsic properties of this semiconductor still represent a latent issue for high-scale applications, because of the fast recombination of the photogenerated charges (electron–hole pairs) during the photocatalytic process and its inability to absorb light in the visible region of the electromagnetic spectrum [1,2]. As an alternative to overcome these problems, different researchers worldwide have studied the doping of TiO_2_ with metallic species such as Ag, Au, Co, Cu, Cr, Fe, Ni, Mn, Pt, and Ru [3], among many others. The modification of titania and other semiconductors with different metals led to improve photochemical properties by acting as electron traps, thus, avoiding the electron–hole pair recombination, reducing the bandwidth, or adding new energy levels to efficiently absorb visible light from the electromagnetic spectrum [4,5,6].

The metalized titania has been useful in heterogeneous photocatalysis since this process allows the use of a clean energy source such as sunlight in the elimination of complex pollutants [7,8].

Heterogeneous photocatalysis is based on the use of a solid semiconductor irradiated by UV-Vis light with an energy higher than or equal to the energy of the band gap (Eg) of this material, and this irradiation produces the excitation and subsequent migration of electrons present in the valence band (VB) to the conduction band (CB), thus generating positive vacancies (h^+^) and electrons (e^−^); these charged species can participate in redox reactions. During the photocatalytic process, reactive oxygen species (ROS) [9] can also be produced, which are represented by hydroxyl radicals, peroxy radicals, and/or superoxide radicals. Due to its high oxidative power, the ROS are responsible for the pollutant degradation and also for the inactivation of pathogenic microorganisms [10].

The use of monometallic photocatalysts has been extensively studied worldwide; however, the evaluation of composites which include two of more metals currently represents an interesting alternative for the environmental remediation process. Focused on this overview, in this research, Ag or Pd were deposited on Cr-TiO_2_ material. These composites were tested in the degradation of the commercial Acid Orange 7 dye (AO7) and in the treatment of real water samples taken from a natural water source, which was contaminated with effluents coming from anthropogenic activities.

## 2. Materials and Methods

### 2.1. Materials

**Figure 1:**
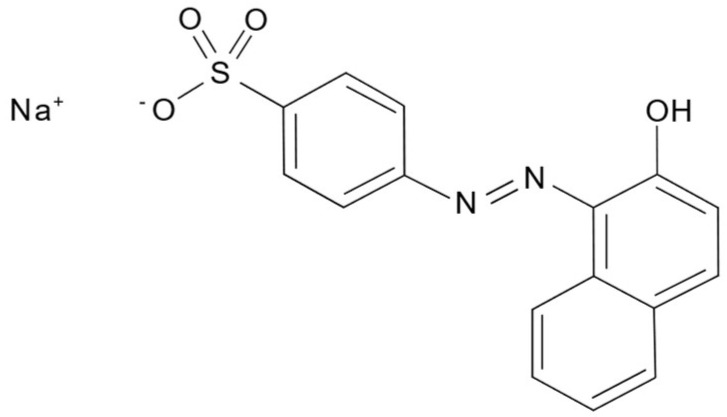
Molecular structure of AO7.

Titanium tetraisopropoxide (C_12_H_28_O_4_Ti > 97% (*w*/*w*) Sigma Aldrich, Milan, Italy), chromium (III) nitrate (Cr(NO_3_)_3_·9H_2_O ≥ 99%), Acid Orange 7 (C_16_H_11_N_2_NaO_4_S 99.95%, Sigma Aldrich), Tetraamminepalladium(II) nitrate solution (Pd(NH_3_)_4_(NO_3_)_2_, 10 wt.% in H_2_O, 99.99%, Sigma Aldrich), Silver Nitrate (AgNO_3_, 99%, Sigma Aldrich), and distilled water (Carlo Erba, Milan, Italy) were purchased and used as received. The molecular structure of AO7 is reported in Figure 1.

### 2.2. Preparation of Cr-TiO_2_ Nanoparticles

TiO_2_ and visible light active Cr-TiO_2_ photocatalyst were prepared by a modified sol-gel method used in our previous work [11]. The Cr/Ti molar ratio used for the preparation of Cr-TiO_2_ was equal to 0.0188, and corresponded to an optimized catalyst formulation. The nominal Cr content was 0.7 wt.%.

It must be remarked that Cr should be included in the titania structure in order to promote the shifting toward the visible region of TiO_2_ optical absorption. This is due to the introduction of intra-band gap states.

### 2.3. Preparation of Pd/Cr-TiO_2_ and Ag/Cr-TiO_2_ Photocatalysts

In a 250 mL flask were added 6.25 mL of isopropanol as a sacrificial agent, the necessary amount of the metal precursor to obtain 0.25% of Ag or Pd over Cr-TiO_2_ particles, and the remaining amount of distilled water [12].

The obtained suspension was poured into a beaker with the necessary mass of photocatalyst and kept under sonication for 10 min in an ultrasonic bath, with a power of 99% and at 20 °C. Subsequently, the suspension was placed in a crystallizer and stirred for 10 min under a nitrogen atmosphere. Then, the suspension was irradiated for 2 h using two 8 W UV lamps, each with a light intensity of 30 mW cm^−2^, under continuous stirring and N_2_ flux. Finally, the suspension was centrifuged, and the solid obtained was dried at 90 °C for 8 h [12]. Noble metal nanoparticles were added to improve the separation of photogenerated charges, owing to their electron-withdrawing ability.

### 2.4. Photocatalyst Characterization

The synthesized materials were characterized by Raman, SEM-EDX, S_BET_, UV-Vis DRS, TG-DTG-DSC, X-ray Diffraction (XRD), X-ray fluorescence (XRF), and Fourier Transformation Infrared Spectroscopy (FT-IR). The detailed description of the equipment and the experimental conditions used in each analysis are reported as follows:

Raman analyses, in the range between 100 and 1000 cm^−1^, were performed using a Dispersive MicroRaman spectrometer (Invia, Renishaw) provided by a 514 nm laser. The specific surface area (S_BET_) of the samples was obtained using the Costech Sorptometer 1042 analyzer by the volumetric N_2_ adsorption at −196 °C. Before the measurements, the degassing pretreatment of the photocatalytic particles was carried out at 150 °C for 30 min in He flux.

SEM-EDX measurements were performed by a Fei Inspect Microscope. Elemental microanalysis of Pd/Cr-TiO_2_ and Ag/Cr-TiO_2_ were obtained by SEM-EDX mapping technique.

The light absorption characteristics of the synthesized photocatalysts were determined by UV-Visible reflectance spectroscopy (UV-Vis DRS). The reflectance data were collected using a Perkin–Elmer spectrophotometer Lambda 35 provided with an 88 sample positioning holder (Labsphere Inc., North Sutton, NH, USA). The reflectance data were presented as a function of wavelength as Kubelka–Munk values (F(R∞)). The determination of the indirect band gap was obtained by plotting [F(R∞) hν]^0.5^ vs. hν (eV) [13].

Thermogravimetric (TG-DTG-DSC) analysis is a thermal technique which measures the weight change and heat flow in a material as a function of temperature and time, in a controlled environment. TG analysis was carried out in a TG analyzer—Q600, TA Instrument, on powder samples under airflow of 100 STP mL/min and with a heating rate of 10 °C/min in a temperature range of 25–900 °C.

The chemical composition of the photocatalytic materials was determined by XRF, using Panalytical Minipal 2 equipment. The experimental parameters used in these analyzes were: He flux, 20 KV and 180 s.

XRD analysis was performed in a Xpert pro Panalytical diffractometer, using the Cu Kα radiation (35 mA and 40 KV).

Fourier transform infrared spectroscopy (FTIR) analyses were carried out using a Thermo Scientific Nicolet TM iS50 FT-IR spectrophotometer, analyzing the samples at a wave number between 4000 and 1000 cm^−1^ with a resolution of 2 cm^−1^, placing the photocatalysts in an ATR cell.

### 2.5. Photocatalytic Activity Test in the AO7 Discoloration

The Pyrex cylindrical tank, used as a photoreactor for assessing the photocatalytic activity of the prepared materials had an internal diameter of 10 cm and a height of 6 cm. During the experimental activity tests, the photocatalyst was kept suspended in the mixture using a magnetic stirrer. A cooling fan was placed close to the photocatalytic reactor to prevent the reaction temperature from rising above 35 °C.

**Figure 2:**
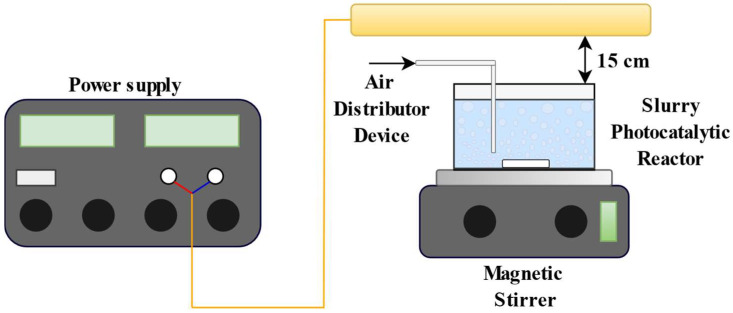
Schematic picture of the experimental setup.

The studies were performed using suspensions with a total volume of 100 mL that contained a fixed initial concentration of dye and a specific photocatalytic nanoparticle dosage. Moreover, the initial pH of the solutions was equal to 5.5, without further pH adjustments. In order to achieve the adsorption–desorption equilibrium of AO7 on the photocatalyst surface, the photoreactor was kept in the dark for two hours before irradiation. Two visible light lamps (Beghelli, Italy) with an irradiance of 30 mW cm^−2^ and a wave-length emission in the range of 400–800 nm were used to illuminate the reactor. The lamps were placed 15 cm above the upper surface of the batch reactor (Figure 2), a reflective metal foil was placed over the photoreactor to limit the dispersion of the visible light irradiations.

About 3 mL of the suspension was taken out of the photoreactor at regular time intervals and then centrifuged to separate the photocatalyst particles.

Aqueous solution aliquots were examined by a UV-Vis spectrophotometer in a Thermo Scientific Evolution 201 apparatus to track the evolution of the dye degradation reaction. The absorbance value at 485 nm was used to analyze the dye concentration [14].

Furthermore, total organic carbon (TOC) content analyses were employed to evaluate the mineralization of the selected contaminant during the photocatalytic tests. In particular, the CO_2_ produced by the high temperature (680 °C) catalytic combustion of the withdrawn samples was used to calculate the TOC reduction of the solution for the different time intervals [15].

A kinetic study of the photocatalytic discoloration of AO7 was also attempted. The kinetics of the photocatalytic process are typically described using the Langmuir–Hinshelwood model [16,17], for which the photodegradation rate (*r*), is expressed as follows:

(1) $$r=\frac{dc}{dt}=\frac{k_{r}K_{ad}\;c}{1+K_{ad}\;c}$$

where *k_r_*, *K_ad_*, and *c* are the kinetic constant for AO7 photodegradation, adsorption equilibrium constant, and dye concentration, respectively.

Equation (1) can be reduced to the first-order kinetics expression with an apparent degradation kinetic constant (*k_app_*) assuming the adsorption is weak, and the compound concentration is low:

(2) $$\ln\left(\frac{c_{0}}{c}\right)=k_{r}\;K_{ad}\;c=k_{app}\;t$$

The slope of the line formed by plotting ln(*c*_0_/*c*) vs. time *t* provides the apparent discoloration kinetic constant value. Moreover, the following relationship was used to calculate the TOC removal (mineralization) (Equation (3)) and AO7 discoloration efficiency (Equation (4)) at a given irradiation time:

(3) $$TOC\;removal\;efficiency\left(t\right)=\left(1-\frac{TOC\left(t\right)}{TOC_{0}}\right)100$$

(4) $$AO7\;discoloration\;efficiency\left(t\right)=\left(1-\frac{c\left(t\right)}{c_{0}}\right)100$$

where *TOC*(*t*) is the total organic carbon at the generic irradiation time (mg L^−1^), *TOC*_0_ is the initial total organic carbon (mg L^−1^), *c*(*t*) is the AO7 concentration at the generic irradiation time (mg L^−1^), and *c*_0_ is the initial AO7 concentration (mg L^−1^).

### 2.6. Photocatalytic Activity Test in the River Water Treatment

For this study, a water sample taken from a Colombian river that is contaminated by industrial and domestic wastewater (geographic coordinates 5.553981, −73.350224) was employed.

The sample was collected following the Standard Methods for the Examination of Water and Wastewater [18]. After sampling, the river water samples were analyzed by different physicochemical methods. To determine the water quality control parameters, different analyses such as Chemical Oxygen Demand (COD), chlorides, nitrates, and total hardness were carried out. These analyzes were performed in a Spectroquant^®^ Move 100 instrument. In order to ensure the reproducibility of the results, each assay was performed twice.

The microbiological analysis was carried out by membrane filtration method Merck SM 9222B and by ISO 9308 method part 1 [18,19].

For the coliforms bacteria content measurements, a Chromocult^®^ agar was employed as a culture medium. Bacteria concentration in the samples is reported in this manuscript as CFU (Colony Forming Units)/100 mL.

For the photocatalytic treatment of the river water, a discontinuous batch Pyrex reactor and the procedure previously described in different works for wastewater treatment were used [20]. The reaction parameters employed were: (i) 250 mL of the water sample, (ii) constant stirring, (iii) light source: an Osram Ultra-Vitalux lamp (300 W) with sun-like radiation spectrum in the UVA and UVB, (iv) light intensity: 30 W/m^2^, (v) Photocatalyst dosage: 1 g/L, (vi) Oxygen flow: 0.84 STP L/h, and (vii) total treatment time: 4 h.

The experiments were carried out under UV-Vis and visible light. For the visible light test, a polyester UV filter sheet (Edmund Optics) was used, which showed 99.9% absorbance below 400 nm.

After 4 h of treatment time, the photocatalyst was recovered by filtration and the treated water was analyzed by the physicochemical and microbiological methods previously described in Section 2.4. All photocatalytic tests were carried out twice, with a standard deviation of 0.05. The reported values are estimated as arithmetic average.

## 3. Results

### 3.1. Photocatalysts Characterization Results

#### 3.1.1. Chemical Composition by XRF

**Figure 3:**
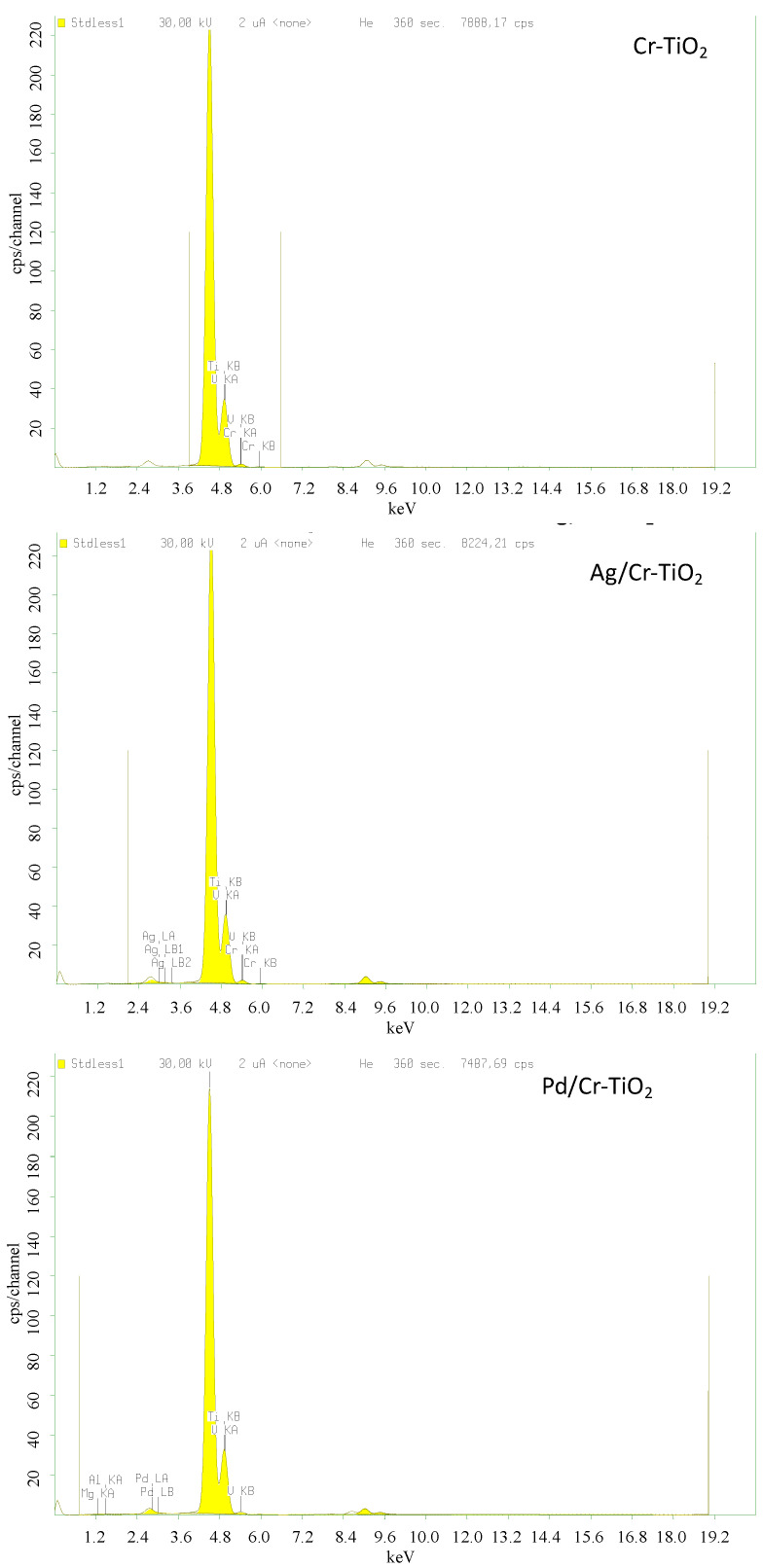
XRF spectra for the photocatalysts analyzed.

The chemical composition of the samples was analyzed by XRF, and, as expected, the presence of Ti, O, Cr, Ag, or Pd was detected in the samples, labelled as Ag/Cr-TiO_2_ and Pd/Cr-TiO_2_ (Figure 3). It is also observed that the Cr content was lower than the nominal one (i.e., 0.6 wt.% versus 0.7 wt.%); however, this could be related to the synthesis method, which brings the Cr within TiO_2_ lattice. The Ag and Pd amount is higher than the nominal content (0.25 wt.%), thus showing the effectiveness of the photodeposition method.

#### 3.1.2. Specific Surface Area Measurement

The surface area measurement was carried out obtaining N_2_ physisorption isotherm at 77 K and by applying the BET theory. The pretreatment of the samples was performed in helium flow at 150 °C for 30 min. Table 1 shows the values of the specific surface area results for the samples.

Cr-doped TiO_2_ had a higher value of SSA than the noble metals decorated TiCrOx samples and pristine TiO_2_, with a result of 113 m^2^/g. This can be explained by considering that the photodeposition of the noble metal leads to the occlusion of the smallest mesopores of the Cr-TiO_2_, reducing the internal surface of the photocatalyst.

#### 3.1.3. UV-Vis DRS Spectra

**Figure 4:**
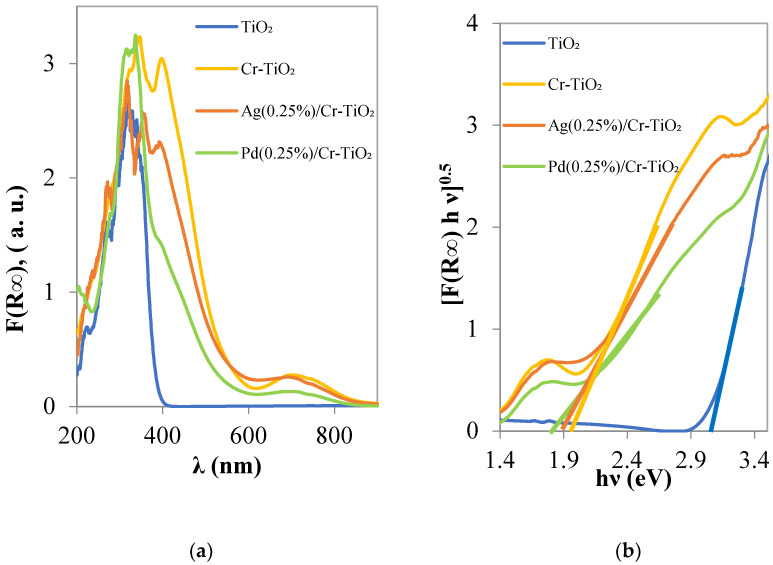
(**a**) K-M spectra for TiO_2_, Cr-TiO_2_, Ag(0.25%)/Cr-TiO_2_, and Pd(0.25%)/Cr-TiO_2_ photocatalysts; (**b**) Band gap calculation by UV–Vis DRS spectra TiO_2_, Cr-TiO_2_, Ag(0.25%)/Cr-TiO_2_, and Pd(0.25%)/Cr-TiO_2_ photocatalysts.

The optical properties obtained by UV-Vis DRS are shown in Figure 4, which were converted to Kubelka–Munk units (Figure 4a) and evaluated with Tauc plot (Figure 4b). TiO_2_ UV absorption has an onset at 400 nm. The presence of Cr into TiO_2_ induced a strong absorption around 405 nm with the onset around 600 nm, indicating the redshift of TiO_2_ absorption in the visible range, which decreases upon the addition of Pd or Ag. Additional absorption bands with respect to TiO_2_ in the range 600–800 nm are observed for Cr-TiO_2_ and Pd or Ag/Cr-TiO_2_. Such absorptions are assigned to the ^4^A_2g_ → ^4^T_2g_ d–d transition of Cr^3+^ in the TiO_2_ structure. The absorptions of charged and metallic clusters, namely Ag_n_^δ+^ and Ag°_m_ where n and m are low numbers, respectively, occurred between 240 and 280 nm, and between 280 and 350 nm, respectively; these absorptions can be observed at 277 and 316 nm in Figure 4a [21]. Moreover, Pd(0.25%)/Cr-TiO_2_ presents an additional band located at 336 nm.

Silver nanoparticles present a broad and very large optical response (surface plasmon resonance, SPR) in the visible region according to their size, with a maximum at 575 nm [22]. It is possible to observe on an Ag/Cr-TiO_2_ sample a slight increase at around 624 nm with respect to Cr-TiO_2_. For Pd/Cr-TiO_2_, a similar trend was observed, even if the d-d transition of Cr^3+^ decreases.

The main decrease in band gap energy of TiO_2_ can be attributed to Cr doping, with a shift from 3.05 to 1.96 eV. The addition of Pd induces a slight decrease in the band gap (BG) value; which in comparison with the sample Ag (0.25%)/Cr-TiO_2_ is more consistent, resulting in 1.85 and 1.90 eV, respectively.

#### 3.1.4. Raman Analyses

**Figure 5:**
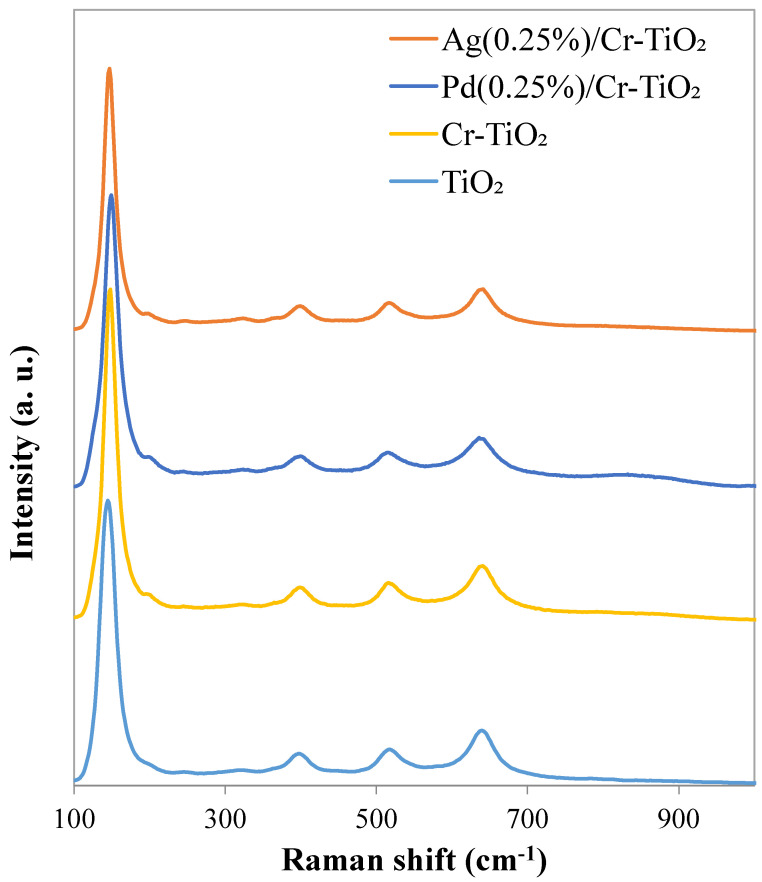
Raman spectra TiO_2_, Cr-TiO_2_, Ag(0.25%)/Cr-TiO_2_, and Pd(0.25%)/Cr-TiO_2_ photocatalysts.

Raman spectra are reported in Figure 5. Main Raman bands, located at 144, 404, 526, and 645 cm^−1^ along with a weak shoulder at 201 cm^−1^, were observed in the spectrum of TiO_2_, which indicates that anatase is the major crystal structure in this material. Indeed, according to the Group theory, tetragonal anatase TiO_2_ presents several Raman active modes: (i) three E_g_ (centered at around 147, 198 and 640 cm^−1^), attributed to symmetrical stretching of O-Ti-O bonds in TiO_2_ NPs; (ii) two B_1g_ at around 400 and 515 cm^−1^, again due to O-Ti-O symmetrical stretching vibrations; (iii) one A_1g_ centered a 514–522 cm^−1^ related to the anti-symmetrical bending vibration [23]. The absence of additional modes, at 447 and 610 cm^−1^, related to E_g_ and A_1g_ modes of rutile phase TiO_2_ indicates that this phase was not formed in the powders [24].

Some weak bands centered at around 240, 287, 324, and 367 cm^−1^ were observed along with main Raman modes of anatase TiO_2_. Brookite A_1g_ vibration mode can be individuated, having seven A_1g_ modes Raman allowed [25] at 125, 152, 194, 246, 412, 492, and 640 cm^−1^, in addition to two missing modes of this symmetry that can also be observed at 324 and 545 cm^−1^. The most intense bands are located at 152, 246, and 545 cm^−1^, so the weak signal at 240 cm^−1^ on our samples, of very low intensity, can be attributed to traces of brookite. Meanwhile, the other main bands are covered by peaks characteristic of anatase phase. Interestingly, a very weak band present at 324 cm^−1^ can be attributed to brookite traces [25].

The remaining two modes centered at around 287 and 367 cm^−1^ are not attributed theoretically to any fundamental modes of TiO_2_. However, it is retained that such broad bands are due to the presence of disorder and defects in TiO_2_ [23,24,26].

The Cr-doped TiO_2_ sample presents at 146 (𝐸_𝑔_), 400 (𝐵_1𝑔_), 518 (combination of 𝐴_1𝑔_ and 𝐵_1𝑔_), 645 (𝐸_𝑔_) and one weak band at 202 cm^−1^ (𝐸_𝑔_), in a similar way to the anatase TiO_2_ s Raman bands [23]. The bands related to the formation of Cr_x_O_y_ species are absent or near to the detection limit, corroborating the hypothesis of Cr introduction into TiO_2_ lattice [11].

On the other hand, noble metals decorated TiCrOx materials samples show similar Raman bands with respect to TiO_2_ Nps and Cr-doped TiO_2_, without additional Raman active modes. Metallic Pd^0^ has no active Raman modes [27]; however, it is widely recognized that Pd nanoparticles can interact with environmental oxygen to form a layer of surfacial PdO [26]. The peaks located at 424 and 275 cm^–1^, expected in the presence of PdO [28], are not discerned in the Pd(0.25)/Cr-TiO_2_ material. The very broad band starting from around 790 cm^−1^ up to about 950 cm^−1^ should be assigned to the Pd photoaddition. On the contrary, Ag nanoparticles are more stable against ambient oxygen oxidation and no signal of metallic silver is found [29]. From the analyses, it can be deduced that all the samples formulated contain crystalline TiO_2_ and are in the predominant form of anatase crystalline phase with the presence of small traces of brookite.

#### 3.1.5. Thermogravimetric Analyses

**Figure 6:**
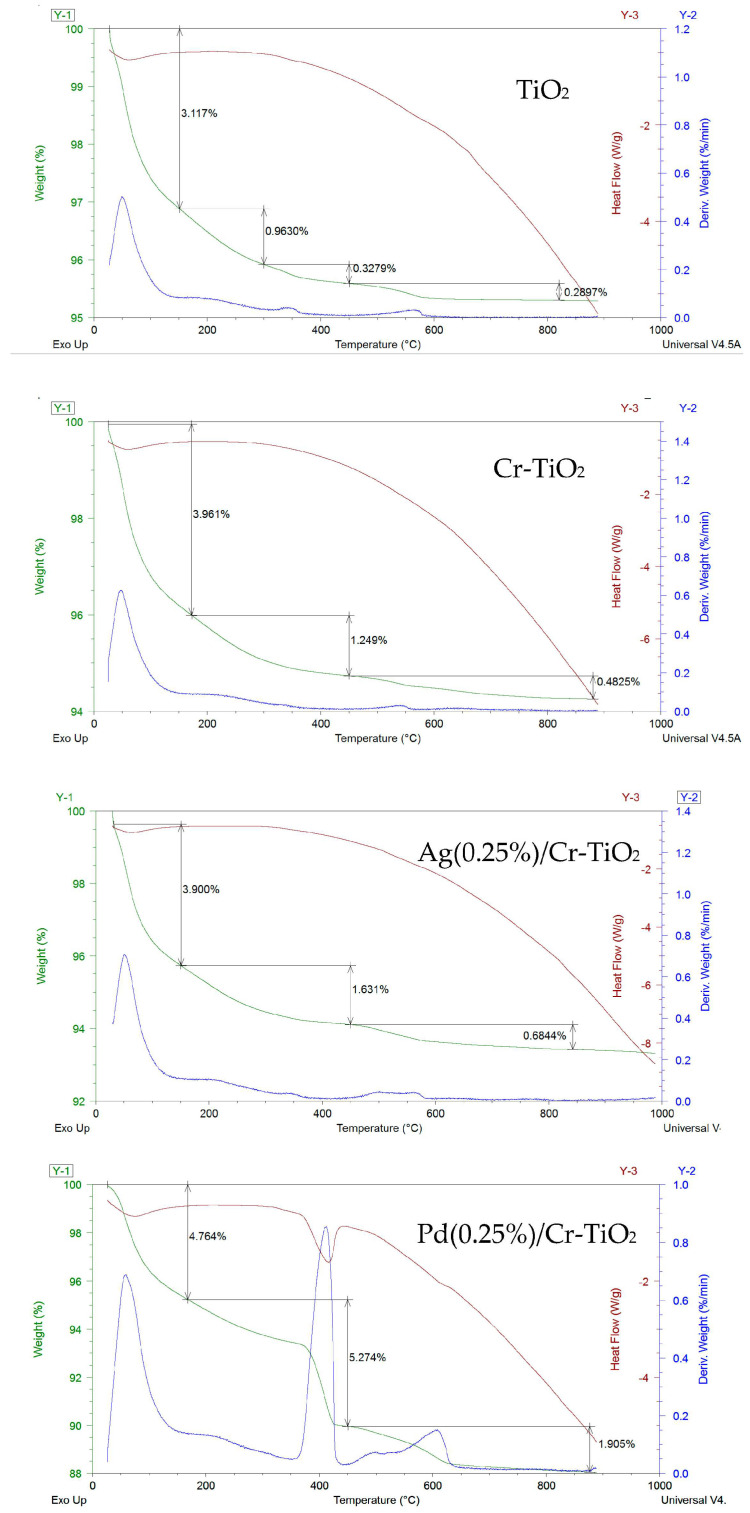
TG-DTG-DSC for TiO_2_, Cr-TiO_2_, Ag(0.25%)/Cr-TiO_2_, and Pd(0.25%)/Cr-TiO_2_ photocatalysts.

The TG-DTG-DSC analyses in Figure 6 show three main steps of weight loss, similar among the samples. It must be remarked that the second loss on TiO_2_ can be divided into two steps, as reported in the relative thermogram, and that TiO_2_ and Cr-TiO_2_ were calcined at 450 °C, so all the endothermic weight losses up to this temperature should be attributed to the interaction with ambient humidity when cooled.

Free water is desorbed from TiO_2_ between 20 and 180 °C, as can be verified by the presence of a DTG and endothermic DTA peak in that region. The amount of this loss is variable, being in the range from 3 to 4.8% for TiO_2_, Cr-TiO_2_, Ag(0.25%)/Cr-TiO_2_, and about 1% for Pd(0.25%)/Cr-TiO_2_.

The second endothermic event that can be individuated from the samples occurs between 180 and 450 °C and, this was associated with the cross-link bonding among the terminal and bridged hydroxyl groups on the surface to form and desorb water.

The water losses associated with that event increased from 1.25–1.29% to 1.63% on Ag(0.25%)/Cr-TiO_2_, and about 5.28% for Pd(0.25%)/Cr-TiO_2_, indicating that some precursor and sacrificial agent could be already present on noble metals decorated TiCrOx materials, which remain, however, to a major extent on Pd(0.25%)/Cr-TiO_2_.

The third exothermic event for the pure and titanium-doped sample is located at 450 °C and up to about 820 °C, probably associated to the loss of further isolated hydroxyl due to aggregation of nanoparticles up to the transition to rutile phase, expected at around 600 °C [30]. It must be remarked that the anatase to rutile transformation is not well defined, but it is time-dependent due to its reconstructive nature.

The third weight loss becomes more complex on noble metals decorated TiCrOx samples and two convoluted contributions can be evinced by DTG: the first in a temperature range comparable with those of TiO_2_ and Cr-TiO_2_, the second in a lower temperature range for noble metals decorated TiCrOx. This latter thermal event occurs with low intensity on Ag(0.25%)/Cr-TiO_2_, and to a greater extent on Pd(0.25%)/Cr-TiO_2_. The latter phenomenon could be attributed to the loss of oxygen of PdO nanoparticles, formed during air exposure during the thermal analysis.

#### 3.1.6. XRD Analyses

**Figure 7:**
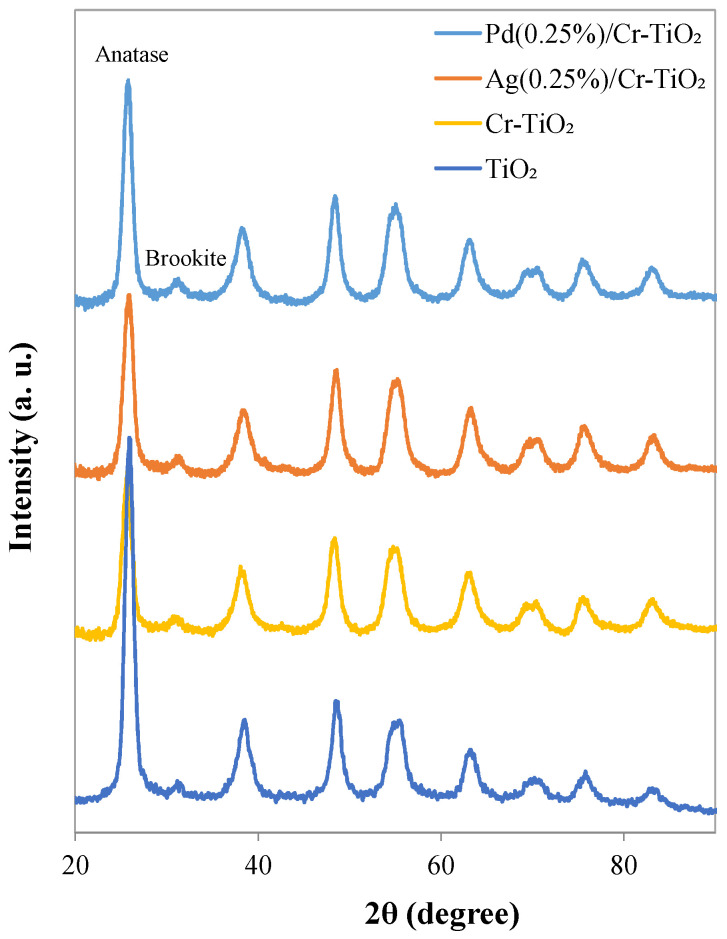
XRD patterns for Cr-TiO_2_, Ag(0.25%)/Cr-TiO_2_, and Pd(0.25%)/Cr-TiO_2_ materials.

XRD diffraction patterns for the studied photocatalysts are shown in Figure 7. The TiCrOx materials modified by impregnation and photochemical reduction present the characteristic peaks of the anatase phase with the main signal located at 2θ = 25.78° [31]. It was also possible to observe a typical signal associated with the brookite phase located at 2θ = 31.12° [32], and the absence of the rutile crystalline phase, which is in good agreement with the results obtained by Raman analysis, as previously described.

Moreover, the diffraction patterns did not show the presence of chromium titanates [33] or chromium oxide [34], which confirms the possible presence of Cr cations in the TiO_2_ lattice. In the case of Cr-based photocatalysts, the Ti^4+^ ion can be successfully substituted by Cr^3+^ since the Ti^4+^ ion has a similar ionic radius (0.68 Å) to the Cr^3+^ ion (0.69 Å), which favors the incorporation of Cr ions into the lattice. However, no significant changes in crystallite size are evident (Table 2) [35]. Nor is it possible to see characteristic signals of the noble metals deposited on the surface; it is mainly due to the low loading of these elements in the synthesized materials.

#### 3.1.7. SEM—EDX Images

**Figure 8:**
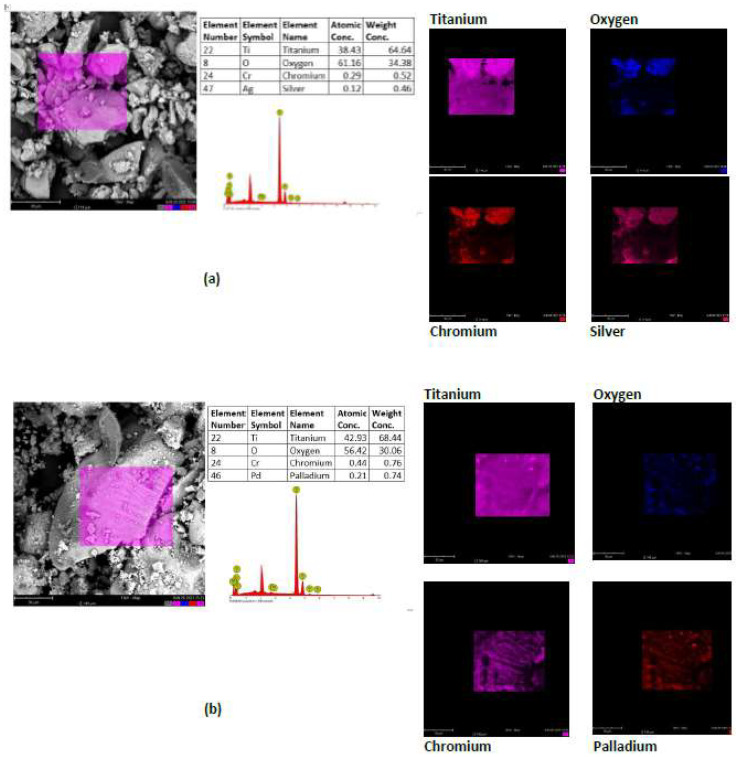
(**a**) SEM-EDX spectra for Ag(0.25%)/Cr-TiO_2_, photocatalyst, and (**b**) Pd(0.25%)/Cr-TiO_2_.

FESEM images of TiO_2_ and Cr-TiO_2,_ which were previously reported in [11], showed the presence of particles mainly composed of aggregated nanocrystallites of about 9 ± 1 nm, almost homogeneous at the nanometer scale, not influenced by the Cr doping. In order to appreciate the distribution of noble metals on a larger scale, SEM-EDX analysis was performed in this work on Ag(0.25%)/Cr-TiO_2_ and Pd(0.25%)/Cr-TiO_2_ photocatalysts. SEM images (Left side Figure 8a,b) show the occurrence of big aggregates together with dispersed small sub-micron particles for both photocatalysts. EDX analyses evidence the presence of Ti and O in smaller amounts with respect to TiO_2_ stoichiometry while the amount of detected Cr is defective for Ag(0.25%)/Cr-TiO_2_ (Figure 8a) and is close to the theoretical nominal amount for the Pd(0.25%)/Cr-TiO_2_ photocatalyst (Figure 8b).

The addition of Ag and Pd dispersed on the surface is evident on Cr-doped TiO_2,_ on the right side in Figure 8. The metals appear well distributed all over the focused sample surface. However, the amounts exceed the nominal load, appearing concentrated in some parts.

#### 3.1.8. ATR-FTIR Spectra

**Figure 9:**
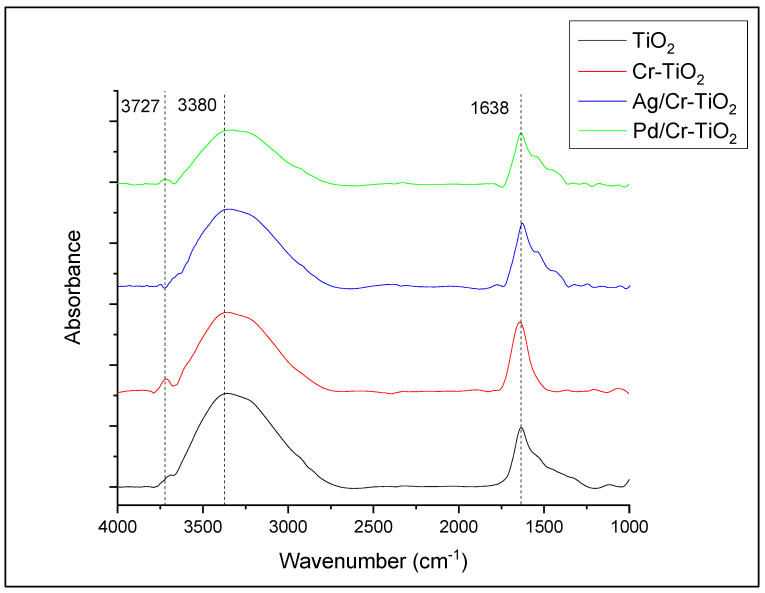
FTIR spectra for TiO_2_, Cr-TiO_2_, Ag(0.25%)/Cr-TiO_2_, and Pd(0.25%)/Cr-TiO_2_.

Figure 9 shows the FTIR spectra of the employed photocatalysts. As it can be observed in this figure, a signal located near 3700 cm^−1^ is evident in all the analyzed samples, which corresponds to the isolated hydroxyl groups. This signal presents the highest intensity in the Cr-TiO_2_ material; after Ag or Pd photodeposition, this band significantly decreases. In the IR region between 2900 and 3600 cm^−1^, there is a wide signal which can be identified as linked surface hydroxyl groups; these groups are located in the more uniform zone of the surface, where there is a regular distribution of titanium and oxygen atoms in the crystalline lattice. In this IR region terminal, Ti-OH species and adsorbed water Ti-OH_2_ can be identified [33,36,37].

The changes observed in the intensity and width of the bands located between 4000 and 2700 cm^−1^ are probably due to the following: (i) By the photodeposition method it is possible to obtain a big number of metal nanoparticles homogeneously distributed on the TiO_2_ surface (as it was observed by SEM-EDX analysis), thus reducing the surface hydroxylation of the Cr-TiO_2_ material after chemical phtoreduction of Ag or Pd [37,38]; (ii) in the case of the Cr-TiO_2_ sample, this presents a more hydroxylated surface as it was observed by FTIR analyzes. This is because the addition of Cr was carried out in situ during the sol-gel synthesis of TiO_2_, so Cr is located inside the lattice, not on the TiO_2_ surface, as it was probed by Raman and XRD analyses [11].

On the other hand, the signal located at 1638 cm^−1^ corresponds to the bending vibration of H-O-H, assigned to non-dissociated water molecules bound to the material structure or adsorbed water [39,40,41].

### 3.2. Photocatalytic AO7 Removal Results

#### 3.2.1. Comparison among the Photocatalysts Performances

Firstly, screening tests were conducted to identify the photocatalyst exhibiting the highest activity in the decolorization and mineralization AO7 (initial concentration, 10 ppm). The reported materials were the following: TiO_2_, Cr-TiO_2_, Ag(0.25%)/Cr-TiO_2_, Pd(0.25%)/Cr-TiO_2_. The tests were carried out using a photocatalyst dosage equal to 3 g L^−1^.

**Figure 10:**
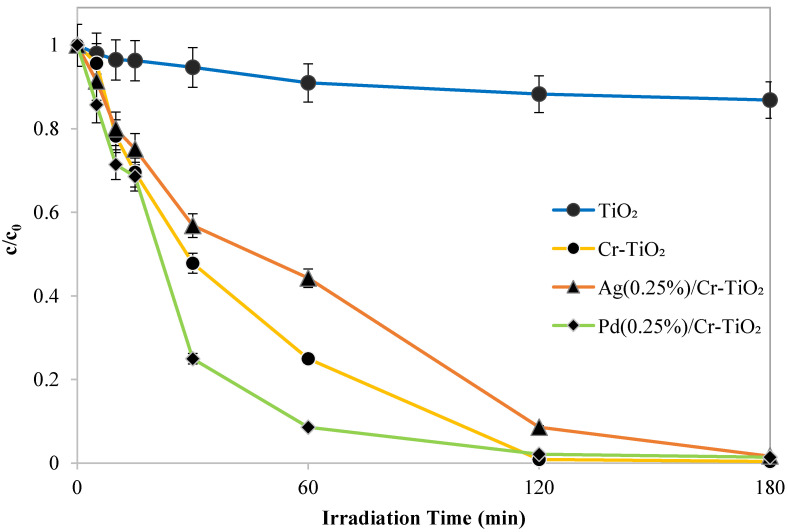
Screening tests TiO_2_, Cr-TiO_2_, Ag(0.25%)/Cr-TiO_2_, and Pd(0.25%)/Cr-TiO_2_: trend of normalized AO7 concentration with respect to its initial concentration as a function of irradiation time.

The results showed that the sample, which effectively allows the degradation and mineralization reactions of the organic dye to accelerate, is Pd(0.25%)/Cr-TiO_2_. As shown in the histogram in Figure 10, it presents the maximum value of the apparent kinetics constant of discoloration of AO7, equal to 0.041 min^−1^, while the samples Cr-TiO_2_ and Ag(0.25%)/Cr-TiO_2_ presented values of 0.0234 and 0.015 min^−1^, respectively.

**Figure 11:**
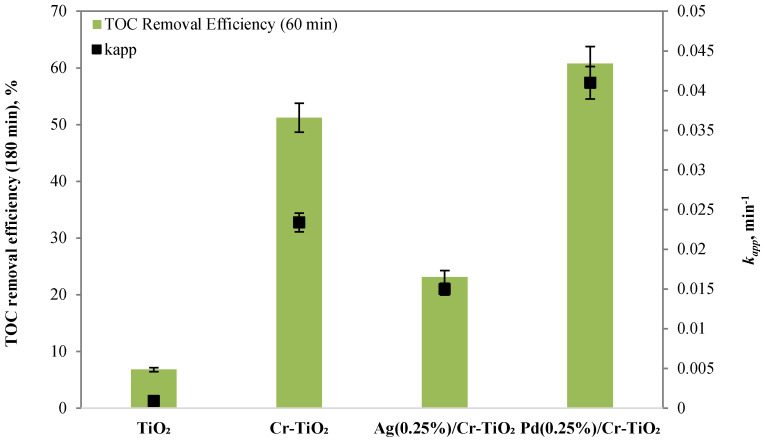
TOC removal efficiency after 3 h under visible light and k_app_ values for the samples obtained by the screening tests of TiO_2_, Cr-TiO_2_, Ag(0.25%)/Cr-TiO_2_, and Pd(0.25%)/Cr-TiO_2_.

Furthermore, the results have shown that all the metal added samples induce the mineralization of the dye, and the Pd(0.25%)/Cr-TiO_2_ sample registered the highest TOC removal efficiency value after 60 min of visible light irradiation (60.8%) (Figure 11). The adopted synthetic strategy to have Cr inside the TiO_2_ structure and noble metals dispersed onto the surface to limit the recombination of e^-^h^+^ pairs was successful in the case of Pd.

#### 3.2.2. Influence of Photocatalyst Dosage

Subsequently, experimental tests were conducted with the sample showing the best performance in the AO7 degradation, i.e., Pd(0.25%)/Cr-TiO_2_.

In particular, the effect of photocatalyst dosage on the AO7 discolouration and mineralization was also examined. Indeed, a proper photocatalyst dosage may improve the photocatalytic performance minimizing the energy cost for the treatment. Indeed, it is a crucial parameter of photocatalytic processes since the number of active sites and photo-adsorption capacity of the catalyst utilized have a significant impact on the photodegradation efficiency [42]. For this reason, the effect of Pd(0.25%)/Cr-TiO_2_ dosage on AO7 photocatalytic degradation was investigated, and the optimal loading value was determined.

**Figure 12:**
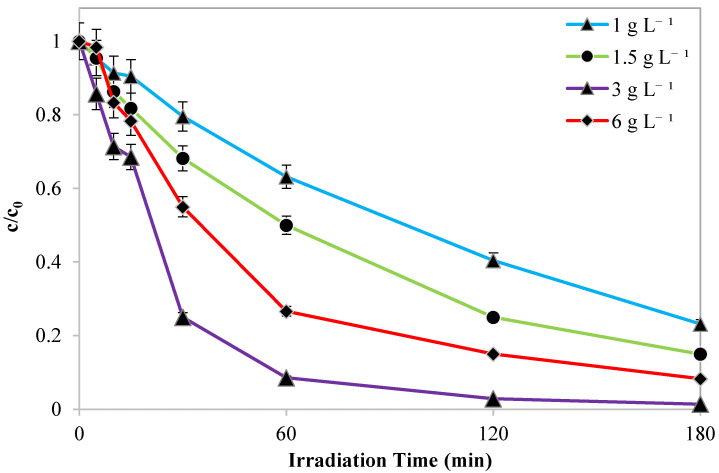
Trend of normalized AO7 concentration with respect to its initial concentration as a function of irradiation time obtained by the different Pd(0.25%)/Cr-TiO_2_ dosage.

**Figure 13:**
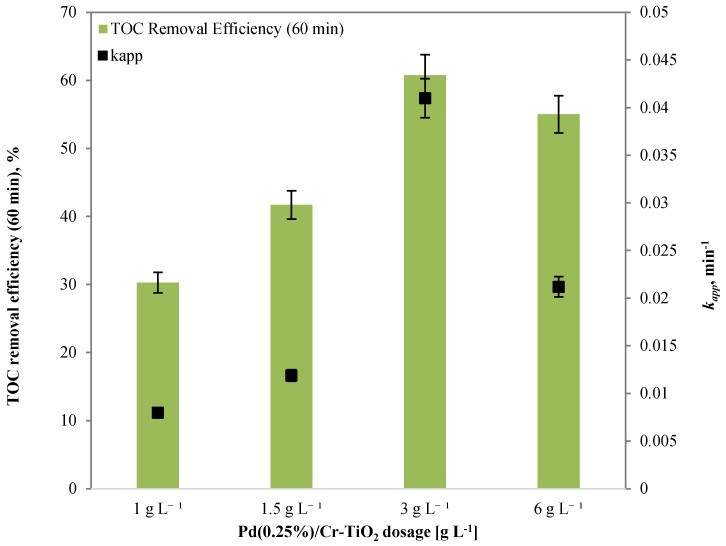
TOC removal efficiency after 3 h under visible light and k_app_ values for the samples obtained by the different Pd(0.25%)/Cr-TiO_2_ dosage.

The Figure 12 and Figure 13 underline that the photocatalyst dosage has a similar influence on AO7 discoloration and TOC removal efficiency. In particular, the results highlighted that the optimal dosage of photocatalyst is equal to 3 g L^−1^. In fact, this dosage allows the maximization of both the discoloration and the mineralization of AO7.

The apparent kinetics constant for the discoloration of AO7 increased from 0.008 min^−1^ to 0.041 min^−1^ as the loading of photocatalytic nanoparticles increased from 1.0 to 3.0 g L^−1^ (Figure 13). Indeed, an adequate increase in photocatalyst dosage raised the number of photons absorbed, which in turn increased the rates of photodegradation [43]. Moreover, photocatalytic degradation and mineralization of AO7 decreased when the loading was changed from 3 to 6.0 g L^−1^. The screening effect of the suspended particles may be the primary reason for the decline in photocatalytic activity. In fact, despite the existence of a large number of active sites, the excessive dosage of the photocatalyst increased the opacity of the solution, reducing the penetration of the photon flux in the reactor, and, thus, decreasing the rate of photocatalytic degradation [44].

#### 3.2.3. Influence of Initial Dye Concentration

**Figure 14:**
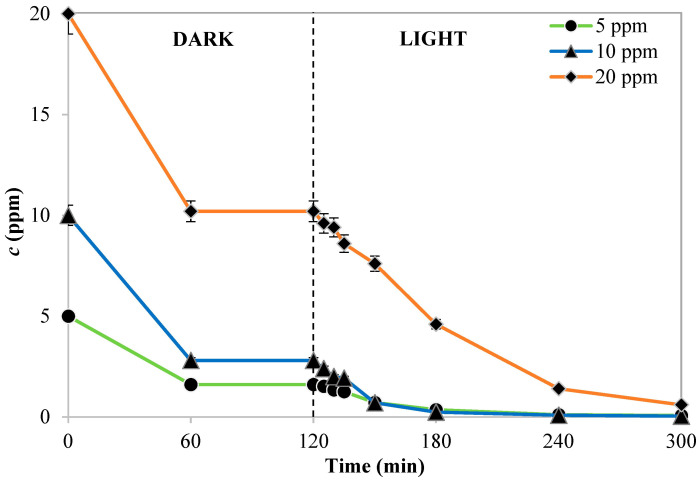
Trend of normalized AO7 concentration with respect to its initial concentration as a function of tests run time obtained by the different Pd/Cr-TiO_2_ dosage.

Next, using the identified optimal photocatalyst dosage (3 g L^−1^), the photocatalytic process at various initial AO7 concentrations was further examined. Figure 14 compares and illustrates the initial relative concentrations of AO7 (c/c_0_) as a function of irradiation time. The data obtained by the experimental tests indicated that 10 ppm is the optimal initial concentration for the photocatalytic reaction. In fact, with an initial AO7 concentration of 10 ppm, the discoloration efficiency of 91.4% occurred after 60 min of visible irradiation. However, the AO7 discoloration efficiency during 180 min of visible light irradiation was 77.8% and 54.9%, at initial concentrations of 5 and 20 ppm, respectively.

In particular, the pollutants mineralization mechanism is due to the action of reactive oxygen species (ROS), such as hydroxyl radicals, superoxide, and positive holes on the photocatalyst surface, and their subsequent interaction with the dye molecules [45]. From the results, presented in Figure 14, it has been shown that the decolorization of the pollutant is comparable for the initial concentrations of 5 and 10 ppm, while it decreases by increasing the initial concentration to 20 ppm. Indeed, by increasing the concentration of the dye excessively, the generation of ROS is disadvantaged because the active sites of the photocatalytic particles are occupied by the adsorbed AO7 molecules [46]. Furthermore, an excessive increase in the concentration of dye in the solution leads to a reduction in the intensity of the light incident on the photocatalyst surface because a greater number of photons of the visible irradiations will be absorbed by the AO7 molecules [47].

#### 3.2.4. Literature Comparison

The apparent pseudo-first order kinetics constants were compared with the literature and are reported in Table 3.

It can be observed that the photocatalyst with the best perforrmance in this work also presents the higher value of kinetics constants.

However, the results obtained in the previously described experimental stages were performed using a lab-prepared dye solution. These outcomes show a great potential as the starting point for potential further applications of photocatalytic materials in environmental remediation processes; however, in order to determine the real potential of these procedures at a big scale, it is also very relevant to test the photocatalytic materials in the treatment of real water samples, where many and different pollutants are present in the same reaction medium. In order to achieve this objective, in the present work the noble metals decorated TiCrOx materials were also evaluated in the river water treatment as described in the next section.

### 3.3. Photocatalytic Tests on Real River Water

#### 3.3.1. Photocatalytic Treatment for River Water under UV Irradiation

The series of photocatalytic materials (TiO_2_, Cr-TiO_2_, Ag(0.25%)/Cr-TiO_2_ and Pd(0.25%)/Cr-TiO_2_) were evaluated in the treatment of river water samples, which are contaminated by anthropogenic activities, such as domestic and industrial work. The results obtained for bacteria removal, as well as other physicochemical parameters, are presented in Table 4.

As shown in Table 4, a slight increase in the pH value was evident after all the treatments applied. It can be due to the in situ production of hydroxyl ions, these ions are responsible for the increase observed in the alkalinity of the liquid phase [52]. This increase in the pH value can have a positive effect, as several researchers have reported higher photocatalytic efficiency at basic pH values [53,54]. It was also observed that the values of total hardness, chlorides, and nitrates slightly decreased after the photocatalytic treatment. This can be attributed to the adsorption of ionic species such as ${NO}_{3}^{-}$, ${Cl}^{-},$ and ${CO}_{3}^{2-}$ on the surface of the photocatalytic materials, thus reducing the pollutant concentration in the fluid phase [20,55].

On the other hand, the chemical oxygen demand (COD) slightly increased after treatment; this behavior can be explained mainly due to the partial oxidation of organic matter present in the water sample. This oxidation can contribute to the production of short chain and more soluble organic compounds. In addition, the broken bacteria cell walls led to the release of ions, hydroxylated compounds, and organic acids; all of these substances can contribute significantly to the COD value increasing [56,57,58,59]. It is also important to note that during the photocatalytic treatment, reactive oxygen species (ROS) are generated. Different authors have indicated that these species can also be responsible for the COD value increasing [60,61]; however, in no case did the COD content exceed the Environmental Protection Agency discharge standards (i.e., 250 mg/L).

As it can also be observed in Table 4, the river water sample under study presents pathogenic bacteria such as coliforms and other Enterobacteriaceae. The cromocult agar employed in this work is a chromogenic culture medium that allows the selective differentiation of bacteria based on the resulting coloration after 24 h of incubation. The colorimetric differentiation with this agar can be described as follows: (i) for *E. coli*, the ß-D-glucuronidase and ß-D-galactosidase enzymes break down the X-glucuronide and Salmon-GAL substrates, resulting in a dark blue to violet coloration; (ii) for other coliform bacteria, the ß-D-galactosidase enzyme reacts with the Salmon-GAL substrate resulting in a pink to red coloration; and (iii) non-coliform bacteria appear colorless or bone white to cream-colored [19].

**Figure 15:**
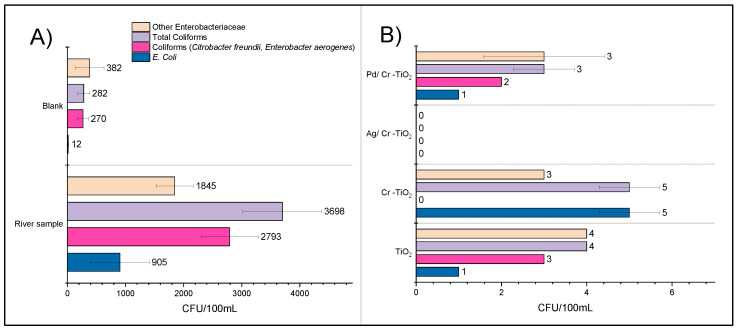
Enteropathogenic bacteria content in the water river sample. (**A**) Before and after blank treatment and (**B**) after photocatalytic treatment.

Figure 15 represents the bacteria content in the river water sample before and after photocatalytic treatment. Firstly, the blank test performed only under UV-Visible light led to a significant decrease of the total coliform bacteria content (i.e., 92.4%); this is mainly due to the widely known antimicrobial effect of UV-Vis light [44], which is the result of the oxidative stress on bacteria cell membrane and intracellular components generated by ROS, as well as of the damage caused to DNA, thus inhibiting the replication of genetic material in the cell [62,63].

As it can be observed in Figure 15, after treatment using the photocatalytic materials under UV-Vis radiation, the bacteria inactivation rate significantly increases, thus achieving a bacteria removal higher than 99% in all the cases. It is important to note that the results obtained using TiO_2_ and Cr-TiO_2_, respectively, are very similar. This can be explained by the characterization results; thus, as it was described in Section 3, the Cr species were found inside the TiO_2_ lattice, not on the surface of this semiconductor, thus reducing the bactericidal effect of the chromium nanoparticles.

On the other hand, using Ag(0.25%)/Cr-TiO_2_ photocatalyst, it was possible to achieve total removal of the bacteria loading from the river water sample. The efficiency trend of the treatments applied in the bacteria elimination was: UV-Vis < TiO_2_ < Cr-TiO_2_ < Pd/Cr-TiO_2_ < Ag/Cr-TiO_2_. In general, the increase observed in the bacteria elimination ratio on TiO_2_ after noble metal addition is mainly due to the bactericidal effect of the metal nanoparticles, but the metallic species can also act as an electron trap during the photocatalytic process, thus increasing the half-time life of the charge carriers, avoiding its recombination. A low recombination rate leads to increase ROS production in the reaction medium [64,65]. The highest bacteria inactivation observed using the Ag/Cr-TiO_2_ photocatalyst can be due to the well-known antimicrobial effect of silver nanoparticles [10,66], which is improved by a synergistic effect between Cr and Ag particles. The Ag/TiO_2_-based materials (from different titania sources) have been extensively studied by different authors worldwide, in terms of total coliform bacteria elimination [10,67,68,69]. In general, the bactericidal effect of silver nanoparticles itself has been widely reported; according to these references, it is expected that Ag/TiO_2_ should be more active than TiO_2_.

The combination of the metals in a noble metals decorated TiCrOx photocatalyst significantly improves the absorption of titania to the visible region of the electromagnetic spectrum, as it was determined by UV-Vis DRS analysis, as previously described. A higher absorption in the UV-Vis region led to improve photocatalytic activity, as it can be observed in the results obtained in the present work.

The coliform bacteria removal by photocatalytic materials from river water that is contaminated by industrial and domestic effluents has been extensively studied by our research group. Thus, by comparison with the results obtained to date (Table 5), we can observe that under the similar experimental conditions of the present work, it was possible to obtain bacteria elimination percentages over 80%. In previous studies, it was only possible to achieve the total coliform bacteria removal using silver or Pt contents in the photocatalysts over 2 wt.%. So, it is very relevant that in this work, using only 0.25% of Ag nominal content coupled to Cr-TiO_2_, the total elimination of *E. coli*, other coliforms (Citrobacter freundii, Enterobacter aerogenes), and other enterobacteriaceae was achieved.

However, in order to ensure the effectiveness of the treatment, and to study the bacterial regrowth, the water samples were maintained outdoor at room temperature for 7 h after treatment. After this time, the samples were analyzed by membrane filtration method and no bacterial regrowth was observed even after 24 h.

**Figure 16:**
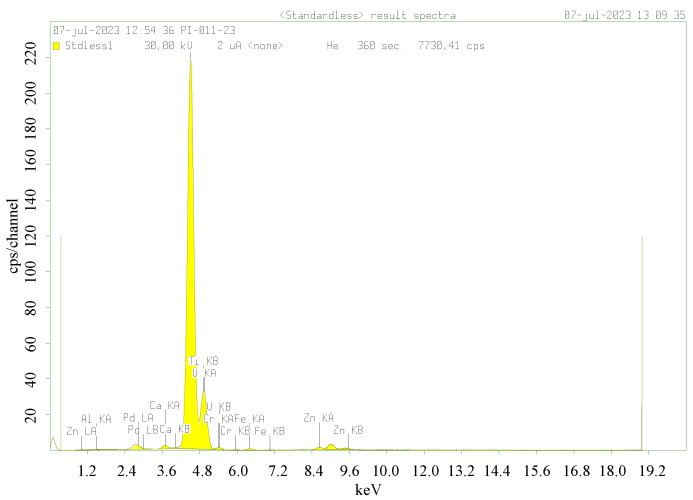
XRF spectra for Pd/Cr-TiO_2_ powder after photocatalytic treatment of the water river sample.

For practical and big scale applications, both high antibacterial activity and low metal leak are two important characteristics for metal-TiO_2_ based materials. In order to analyze the stability of the Pd/Cr-TiO_2_ material (only for reference purposes), after photocatalytic treatment of the water river sample, the recovered material was analyzed by XRF (Figure 16) and it was observed that the Pd and Cr content remain almost the same, only 0.1% of difference before and after treatment was observed. This small difference can be associated with the error expected in the method of analysis. However, it is important to consider this for further application of the photocatalytic materials.

From XRF analysis, it is also important to note that after photocatalytic reaction, the photocatalytic material includes in its composition new elements; these pollutants can come from the water sample.

In order to test the effectiveness of the photocatalytic materials only under visible light, different experiments were performed and the results are presented below.

#### 3.3.2. Photocatalytic Treatment of River Water under Visible Light

After treatment under visible light, a decrease was observed in the concentration of ionic species such as ${NO}_{3}^{-}$, ${Cl}^{-},$ and ${CO}_{3}^{2-}$ (Table 6), similar to that results observed under UV-Vis light. The pH value also increases after treatment.

However, different from the results obtained under UV-Vis, when only visible light is employed in the treatment, the COD decreases. This is probably due to a less effectiveness in the degradation of chemical organic compounds.

**Figure 17:**
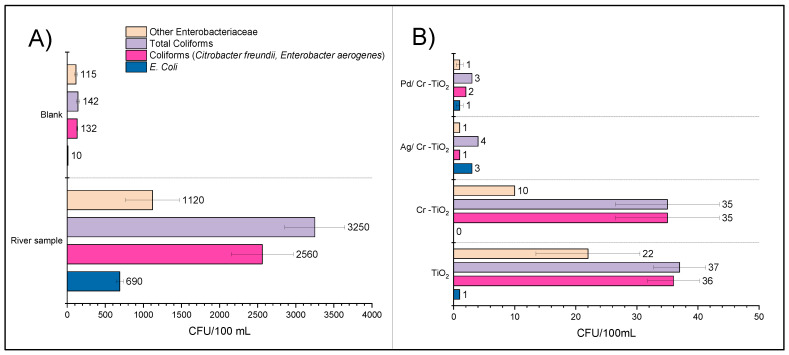
Enteropathogenic bacteria content in the water river sample. (**A**) Before and after blank treatment and (**B**) after photocatalytic treatment under visible light.

Figure 17 represents the bacteria removal under visible light; it can be observed that the treatment using visible light is effective for the elimination of coliform and other enterobacteria. This confirms that TiO_2_, after modification with noble metals, is able to absorb part of the visible spectrum due to an effective electron transfer from the semiconductor conduction band to the metal [71].

In all cases, when using the noble metals decorated TiCrOx materials, better bacterial elimination effectiveness was obtained compared to the use of unmodified TiO_2_ or Cr-TiO_2_, thus demonstrating that the use of two metallic species in the semiconductor can considerably improve its photocatalytic properties.

## 4. Conclusions

Noble metals decorated TiCrOx photocatalysts such as Ag/Cr-TiO_2_ and Pd/Cr-TiO_2_ were successfully obtained by combining the sol-gel method for Cr incorporation and subsequently the photochemical reduction for Ag or Pd addition.

According to the physicochemical characterization of the synthesized material, the Cr incorporation in the TiO_2_ crystalline lattice was evidenced by Raman and XRD, and Ag or Pd decoration of nanoparticles with a homogeneous distribution on the Cr-TiO_2_ surface was shown by EDX analysis.

When studying the photoactivity of the materials, it was determined that the material with the highest decolorization and mineralization activity of the AO7 solution was Pd(0.25%)/Cr-TiO_2_, with an apparent kinetic constant of AO7 decolorization equal to 0.041 min^−1^ when using a catalyst concentration of 3 g/L.

Bacteria elimination tests showed that the materials studied are effective in the reduction of coliform bacteria loading. The experiments showed that the best material using UV-Vis light was Ag(0.25%)/Cr-TiO_2_. When using only visible light, the materials modified with Pd and Ag showed similar effectiveness. In this context, noble metals decorated TiCrOx materials allows the simultaneous treatment of organic and inorganic pollutants, and microorganisms, thus showing a favorable perspective for the treatment of river water highly contaminated by anthropogenic activity.

With both substrates analyzed (i.e., AO7 or river water), the noble metals decorated TiCrOx materials were more effective in the treatment than the effectiveness observed by using Cr-TiO_2_.

Further research could be addressed in order to explore different metal contents in the photocatalytic materials, thus studying its correlation with the bactericidal effect and organic dye degradation.

## Figures and Tables

**Table 1:** Specific surface area (SSA) of the analyzed samples.

Sample	SSA [m^2^ g^−1^]
TiO_2_	107
Cr-TiO_2_	113
Pd(0.25%)/Cr-TiO_2_	106
Ag(0.25%)/Cr-TiO_2_	96

**Table 2:** Anatase crystallite size for the photocatalyst analyzed.

Photocatalyst	Anatase Crystallite Size (nm)
Cr-TiO_2_	7.59
Ag/Cr-TiO_2_	7.26
Pd/Cr-TiO_2_	7.64

**Table 3:** Comparison of photocatalytic performances with literature results under visible irradiation.

Photocatalytic System	Photocatalyst	Type of Light	Type of Azo Dye	*k* [min^−1^]
Our system at the optimal operative conditions	Pd(0.25%)/Cr-TiO_2_	Visible	AO7	0.041
[48]	Pt(0.3%)/S-TiO_2_	Visible	Methyl Orange	0.017
[49]	Cu(10%)/TiO_2_	Visible	Methyl Orange	0.001
[50]	Pt(1%)/TiO_2_	Visible	Reactive Black 5	0.001
[51]	Fe-N-TiO_2_	Visible	AO7	0.03
[47]	Fe-Pr-TiO_2_	Visible	AO7	0.0325

**Table 4:** Water quality control parameters analyzed before and after photocatalytic treatments under UV-Vis radiation.

Quality Control Parameters	Starting River Water Sample	Blank Test UV-Vis	TiO_2_	Cr-TiO_2_	Ag/Cr-TiO_2_	Pd/Cr-TiO_2_
pH	6.10	7.12	6.92	7.13	6.83	6.32
Nitrates (mg/L)	0.63	<0.3	<0.3	0.3	0.3	0.6
Chlorides (mg/L)	24	22	22	21	20	24
Total hardness (mgCaCO_3_/L)	43	32	26	25	29	25
COD (mg/L)	67	75	96	128	151	121
*E. coli* (CFU/100 mL)	905	12	1	5	0	1
Other coliforms (*Citrobacter freundii, Enterobacter aerogenes*) (CFU/100 mL)	2793	270	3	0	0	2
Total Coliforms (CFU/100 mL)	3698	282	4	5	0	3
Other Enterobacteriaceae (CFU/100 mL)	1845	382	4	3	0	3

**Table 5:** Coliform bacteria removal by different photocatalytic materials from river water highly polluted by industrial and domestic effluents.

Coliform Bacteria Loading (CFU/100 mL)	Average Bacteria Elimination (%)
Commercial TiO_2_ (Sigma Aldrich) [12]	98
Faceted TiO_2_ [12]	99
Faceted TiO_2_–Ag 5% [12]	100
Commercial TiO_2_ (P25 Evonic) [20]	40
(0.5%wt.) Pt-TiO_2_ (sulfated) [20]	80
(2%wt.) Pt-TiO_2_ at 120 W/m^2^ (sulfated) [20]	100
Pt-TiO_2_ [70]	99

**Table 6:** Water quality control parameters analyzed before and after photocatalytic treatments under Visible radiation.

Quality Control Parameters	Starting River Water Sample	Blank Test (Visible Light)	TiO_2_	Cr-TiO_2_	Ag/Cr-TiO_2_	Pd/Cr-TiO_2_
pH	6.07	7.09	6.91	7.04	6.35	6.1
COD (mg/L)	68	60.0	39.7	18	26.7	47
Nitrates (mg/L)	0.73	0.6	<0.3	0.4	0.5	0.6
Chlorides (mg/L)	30.5	32.3	27.3	27.3	27.7	27.7
Total hardness (mg CaCO_3_/L)	61.3	63.0	30.7	29.0	40.3	32.3
*E. coli* (CFU/100 mL)	690	10	1	0	3	1
Coliforms (*Citrobacter freundii, Enterobacter aerogenes*) (CFU/100 mL)	2560	132	36	35	1	2
Total Coliforms (CFU/100 mL)	3250	142	37	35	4	3
Other Enterobacteriaceae (CFU/100 mL)	1120	115	22	10	1	1

## Data Availability

Not applicable.
